# Adipose Tissue-Derived Stem Cell Secreted IGF-1 Protects Myoblasts from the Negative Effect of Myostatin

**DOI:** 10.1155/2014/129048

**Published:** 2014-01-23

**Authors:** Sebastian Gehmert, Carina Wenzel, Markus Loibl, Gero Brockhoff, Michaela Huber, Werner Krutsch, Michael Nerlich, Martin Gosau, Silvan Klein, Stephan Schreml, Lukas Prantl, Sanga Gehmert

**Affiliations:** ^1^Applied Stem Cell Research Center, University Medical Center Regensburg, 93053 Regensburg, Germany; ^2^Department of Trauma Surgery, Center of Plastic and Hand Surgery, University Medical Center Regensburg, 93053 Regensburg, Germany; ^3^Department of Trauma Surgery, University Medical Center Regensburg, 93053 Regensburg, Germany; ^4^Department of Obstetrics and Gynecology, University Medical Center Regensburg, 93053 Regensburg, Germany; ^5^Department of Cranio-Maxillofacial Surgery, University Medical Center Regensburg, 93053 Regensburg, Germany; ^6^Department of Dermatology, University Medical Center Regensburg, 93053 Regensburg, Germany

## Abstract

Myostatin, a TGF-**β** family member, is associated with inhibition of muscle growth and differentiation and might interact with the IGF-1 signaling pathway. Since IGF-1 is secreted at a bioactive level by adipose tissue-derived mesenchymal stem cells (ASCs), these cells (ASCs) provide a therapeutic option for Duchenne Muscular Dystrophy (DMD). But the protective effect of stem cell secreted IGF-1 on myoblast under high level of myostatin remains unclear. In the present study murine myoblasts were exposed to myostatin under presence of ASCs conditioned medium and investigated for proliferation and apoptosis. The protective effect of IGF-1 was further examined by using IGF-1 neutralizing and receptor antibodies as well as gene silencing RNAi technology. MyoD expression was detected to identify impact of IGF-1 on myoblasts differentiation when exposed to myostatin. IGF-1 was accountable for 43.6% of the antiapoptotic impact and 48.8% for the proliferative effect of ASCs conditioned medium. Furthermore, IGF-1 restored mRNA and protein MyoD expression of myoblasts under risk. Beside fusion and transdifferentiation the beneficial effect of ASCs is mediated by paracrine secreted cytokines, particularly IGF-1. The present study underlines the potential of ASCs as a therapeutic option for Duchenne muscular dystrophy and other dystrophic muscle diseases.

## 1. Introduction

Duchenne muscular dystrophy (DMD) is caused by mutations in the dystrophin gene and patients suffer from decrease of muscle tissue [1, 2]. By age of 12 most patients are wheelchair-bound and die early between the age of 20–30 due to respiratory failure. The progressive fiber damage and membrane leakage are caused by the destabilized dystrophin-associated protein complex (DAPC) due to the missing dystrophin protein. In fact, a single exposure to high-force eccentric contractions without prior adaptation induce a loss of DAPC components and apoptosis in normal muscle tissue [3, 4]. Normal muscles tissue, repeatedly subjected to mechanical overload, respond with growth and adapting with a greater resistance to membrane wounding [5]. It has been demonstrated that endurance training upregulates IGF-1 and IGF2 mRNA as well as IGF regulatory proteins (IGFBP2, IGFBP4, IGFBP7, and PRSS11). This is of particular importance, since IGF signaling pathway interacts with TFG-b signaling, influencing muscle cell differentiation and proliferation in a complex manner [6]. TGF-*β* protein concentration has been shown to be elevated in dystrophic muscle whereas this protein did not appear in the exercise training analysis for normal muscle tissue [7, 8]. Especially, myostatin, a new member of the TGF-*β* superfamily, has been reported to be a negative regulator of skeletal muscle growth [9]. This would suggest a role of TGF-*β* in the degeneration of skeletal muscles in DMD (e.g., disruption of IGF-related signaling). Indeed, application of IGF-1 attenuates the deterioration of skeletal muscles mass in a murine Duchenne (mdx) model [4, 10].

Previous studies demonstrated beneficial effects of IGF-1 produced by mesenchymal stem cells, particularly by adipose tissue-derived stem cells (ASCs) which can be easily harvested and expanded *in vitro* [11]. Thus, this study was performed to investigate whether paracrine factors secreted by ASCs are able to stimulate proliferation of myoblasts and ameliorate myostatin effects on myoblasts. Moreover, we wanted to elucidate the role of IGF-1 and its protective role for myoblasts when exposed to an elevated myostatin level.

## 2. Materials and Methods

### 2.1. Cell Culture

Adipose tissue was harvested from the inguinal fat pads of five male BALB/c mice (Jackson Laboratory) at the age of 8–12 weeks and pooled for further processing. The tissue was washed twice with prewarmed PBS prior to mincing. Two units of Liberase MNP-S (Roche) were mixed in 1 mL prewarmed Dulbecco's modified eagle medium (DMEM) (Invitrogen) per gram fat and incubated at 37°C 225 rpm shaking for 30 minutes followed by mixing with a pipette to release cells. Afterwards, mixture was centrifuged at 500 g for 10 min and cell pellet was washed three times with PBS. The resulting cell pellet was resuspended and propagated in complete ASCs culture medium (DMEM) supplemented with 20% fetal bovine serum (FBS, PAN-Biotech), 100 U/mL Penicillin (Sigma), and 100 *μ*g/mL streptomycin (Sigma) and incubated at 37°C in a humidified atmosphere containing 5% CO_2_. C2C12 cells (ATCC) were cultured in growth medium consisting of DMEM, 10% FBS, 100 U/mL Penicillin, and 100 *μ*g/mL streptomycin. Myoblasts were subcultured before reaching 80% confluency and used for all experiments before passage 7.

For lentiviral production, human embryonic kidney 293T cells (HEK293T, ATCC) were grown in DMEM containing 10% FBS and used in passages below 20.

### 2.2. Real-Time Fusion Assay

**Figure 1:**
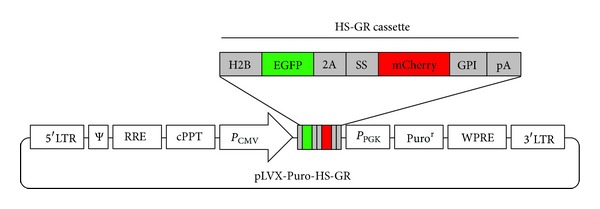
The pLVX-Puro-HS-GR vector was constructed by cloning HS-GR cassette into CMV driven lentiviral backbone (Clontech) between XhoI and XbaI restriction sites. H2B: histone protein sequence; 2A: self-cleavage peptide; SS: endoplasmatic reticulum signal peptide; GPI: C terminal GPI anchor sequence; pA: bovine growth hormone polyadenylation signal.

A plasmid containing H2B-EGFP-2A-mCherry-GPI (HS-GR) was generously provided by Dr. Richard Beheringer and Chuan-Wei Jang (MD Anderson Cancer Center). A lentiviral construct was generated by subcloning the HS-GR cassette into the pLVX-Puro vector (Clontech) between Xhol and Xbal by restriction digest and ligation (Figure 1). The integrity of cloned HS-GR cassette was confirmed by DNA sequencing and resulting vector was named pLVX-Puro-HS-GR.

ASCs were stable transfected by lentiviral infection as described below. DAPI dye was added to myoblast cell culture at a final concentration of 50 *μ*g/mL in 10% FBS supplemented DMEM and incubated for 30 minutes. DAPI labeled myoblasts and pLVX-Puro-HS-GR expressing ASCs cultured in 20% FBS supplemented DMEM were rinsed six times with PBS and trypsinized for coculture fusion assay. 8 × 10^4^ DAPI labeled C2C12 and 2 × 10^4^ pLVX-Puro-HS-GR labeled ASCs were mixed and placed in a 6-well plate and incubated until cell culture reached 100% confluency using myoblast specific medium (DMEM, 10% FBS, 100 U/mL Penicillin, and 100 *μ*g/mL streptomycin). Afterwards, media was switch to fusion media (DMEM supplemented with 2% horse serum) and replaced every second day. Control group of ASC cell culture was propagated in fusion media to monitor spontaneous fusion of cells.

### 2.3. Lentiviral Particle Production and Transduction of ASCs

Commercial available short hairpin RNA (shRNA) constructs were obtained as bacterial glycerol stocks (Sigma) and used to silence murine IGF-I. Plasmid DNA of shRNA and pLVX-Puro-HS-GR vector was purified using a plasmid extraction kit (Plasmid Maxi Kit, Qiagen). Lentiviral vector particles were produced by three plasmid cotransfection onto HEK 293T with 40 *μ*g of shRNA or pLVX-Puro-HS-GR vector DNA, 30 *μ*g pCMV-ΔR8.91 (The Broad Institute, MA, USA), and 10 *μ*g pMD2.G (Addgene, clone 12259) using a calcium-phosphate transfection kit (Invitrogen). Lentiviral vector concentration was determined by p24 ELISA (Cell Biolabs). ASCs were transduced in passage 2 with 2.56 × 10^5^ TU/mL virus and polybrene (8 *μ*g/mL) (Chemicon). After 8 hours, the medium was replaced by fresh DMEM. Twenty-four hours later, antibiotic selection (1.3 *μ*M puromycin) was initiated for 10 days following additional 4-day recovery. ASCs transduced with nontarget shRNA (Sigma) served as negative controls. Stable silencing of IGF-I in ASCs was determined by SYBR Green assay (Biorad) and ELISA (RnD System).

### 2.4. Apoptosis and Proliferation Assay

1 × 10^3^ C2C12 cells were seeded in quintuplicates into a 96-well plate and treated with myostatin after 24 hours resting period followed by further 24 hours incubation. Apoptosis of myoblasts after various myostatin concentrations (RnD Systems) was investigated by Cell Death Detection Kit (Roche) according to instruction manual.

In order to quantify proliferation of murine C2C12 under myostatin treatment cells were seeded at concentration of 1 × 10^3^ in each well of the 96-well plate and proliferation was detected by a BrdU kit (Roche) according to the manufacturer's instruction. In detail, cells were seeded in quintuplicate and allowed to attach for 24 hours followed by myostatin exposure under specific treatment conditions for another 24 hours. Myostatin was added to conditioned medium at indicated final concentration prior to myoblasts incubation. IGF-1 receptors of myoblasts were blocked by adding receptor antibodies (Abcam) 30 minutes prior to myostatin incubation. IGF-1 receptor and IGF-1 neutralizing antibody (RnD System) were simultaneously added to myoblasts in addition to myostatin and incubated for 24 hours.

### 2.5. Conditioned Medium

Conditioned medium of ASCs was used to investigate the effect of ASCs paracrine factors. Serum free DMEM medium containing antibiotics (penicillin, streptomycin) was added to 90% confluent ASCs and collected after 48 h. The conditioned medium was centrifuged at 500 g for 5 minutes and subsequently filtered by a 0.22 micron filter (Millipore). Fresh prepared CM of wild type ASCs, IGF-1 shRNA, and nontarget shRNA transfected ASCs were used for further experiments.

### 2.6. Western Blot

C2C12 cells were seeded in order to obtain 40% confluent cultures prior to adding serum-reduced (2.5% FBS) medium or conditioned medium (CM) containing 0.75 *μ*g/mL myostatin. Total protein content of cell culture was extracted after 72 hours of myostatin incubation when culture reached 70% confluency. First, C2C12 cells were rinsed twice with PBS and trypsinized followed by centrifugation at 500 g for 5 minutes. The cell pellet was washed twice with ice cold PBS subsequently lysed with fresh prepared RIPA-B-buffer and incubated on ice for 15 minutes followed by centrifugation at 13.000 g for 20 minutes at 4°C. Protein concentration was measured by using Bradford method (Dc Protein Assay kit, BioRad) and samples were prepared with Laemmli buffer (BioRad) for SDS-PAGE. Gels were loaded with 90 *μ*g total protein and run for 50 minutes at 100 V. Afterwards, the gel was blotted on a nitrocellulose membrane for one hour at 100 V. The nitrocellulose membrane was blocked in fresh prepared TBS-T/5% milk at room temperature for one hour. MyoD primary antibody (Santa Cruz; sc-71629) was used at a dilution of 1 : 200 and *β*-Actin primary antibody (cell signaling, number 4967) at a dilution of 1 : 1000 and incubated at 4°C overnight. The nitrocellulose membrane was washed four times for five minutes with TBS-T and subjected to appropriate secondary antibody Anti-mouse IgG (DyLight 800 or 680, Cell Signaling) for one hour at room temperature. The Infrared Imaging System Odysee (Licor) was used to obtain digital reading of Western blot.

### 2.7. Real-Time PCR

Total RNA was extracted from cell culture using RNAqueous-Micro-Kit according to manufactures protocol and stored at −80°C until further investigations. Using iScript cDNA Synthesis Kit (BioRad) RNA was reverse transcribed into cDNA according to manufacturer's instructions. To determine MyoD mRNA expression quantitative analyses were performed using Eco qPCR system (illumina) with DyNAmo ColorFlash SYBR Green assay (Biozyme) and appropriate primer set (8 nM final concentration) *β*-Actin and GAPDH primer set served as a housekeeping gene reference (Table 1).

### 2.8. Statistical Analysis

Results are shown as mean ± SD with at least 3 replicates. In addition, all experiments were performed 3 times. For pair wise comparison, a Student's *t*-test was carried out, and for group wise comparison, one-way ANOVA with Scheffe post hoc correction was carried out by using SPSS statistical software package (SPSS Inc., IL, USA). Values at *P* < 0.05 were considered as statistically significant.

## 3. Results

### 3.1. Characterization of ASCs

**Figure 2:**
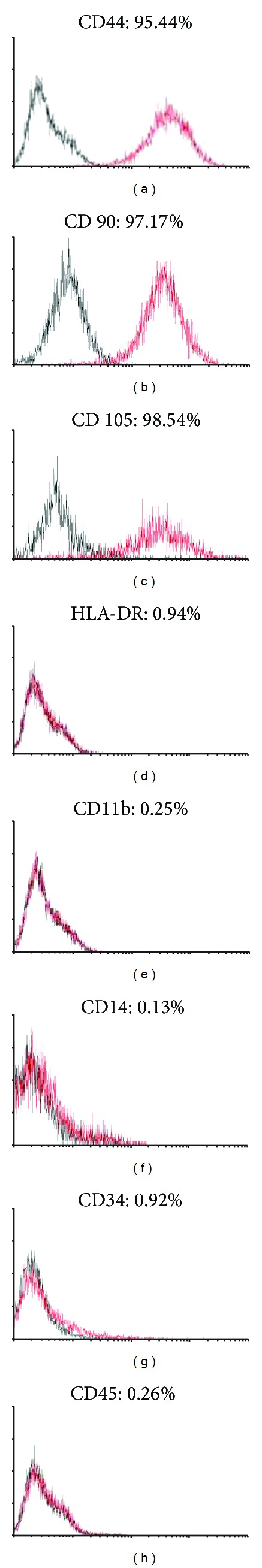
Flowcytometry analyses of ASCs for hematopoietic and endothelial cell markers as well as for mesenchymal cells surface proteins.

Flowcytometry analyses were performed on ASCs for hematopoietic and endothelial cell markers as well as for mesenchymal cells surface proteins. ASCs were positive for CD44 (95.44% ± 2.66), CD90 (97.17% ± 4.05), and CD105 (98.54% ± 1.89) and negative for CD11b (0.25% ± 0.14), CD14 (0.13% ± 0.09), CD34 (0.92% ± 1.51), and CD45 (0.26% ± 0.30), which preclude contamination by hematopoietic cells. HLA-DR, a class II antigen of HLA, was also negative (0.94% ± 0.68) for ASCs (Figure 2).

### 3.2. Fusion of Murine ASCs and Murine Duchenne Myoblasts

**Figure 3:**
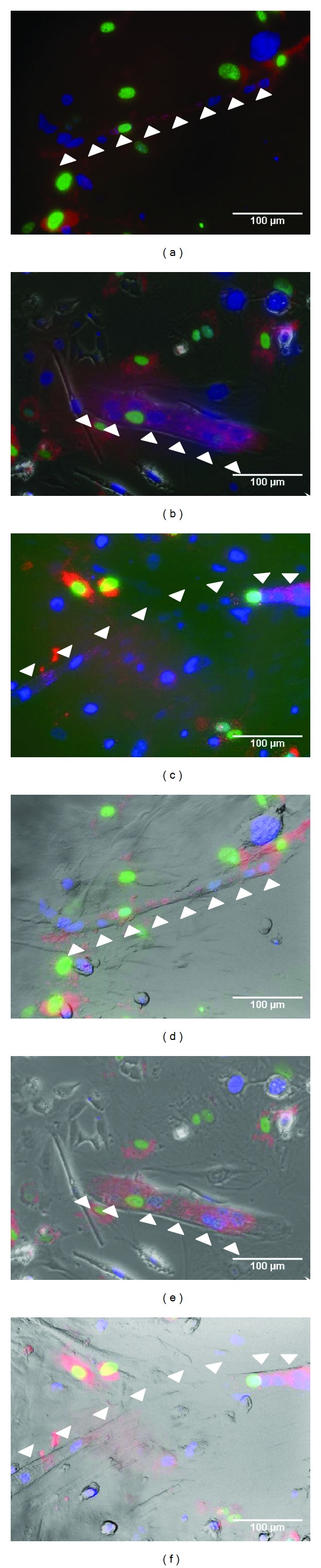
Fusion of DAPI labeled murine myoblasts (blue nucleus) and murine ASCs carrying the pLVX-Puro-HS-GR vector (red cell membrane, green nucleus) monitored by immunofluorescence ((a)–(c)) and phase contrast microscopy overlay ((d)–(f)). The red cell membrane surrounding blue and green nuclei indicates a fusion between myoblasts and ASCs (white arrow indicating myotubes generated by fusion).

DAPI labeled murine Duchenne myoblasts and pLVX-Puro-HS-GR labeled murine ASCs were cocultured and monitored for myotube formation in order to evaluate whether murine ASCs contribute to myotubes by fusion. After 3–5 days DAPI and GFP labeled nuclei were found to be surrounded by a red cell membrane clearly indicating a fusion of both cell types (Figure 3).

### 3.3. Myostatin Induces Apoptosis on Myoblast Cell and Inhibits Myoblast Proliferation

**Figure 4:**
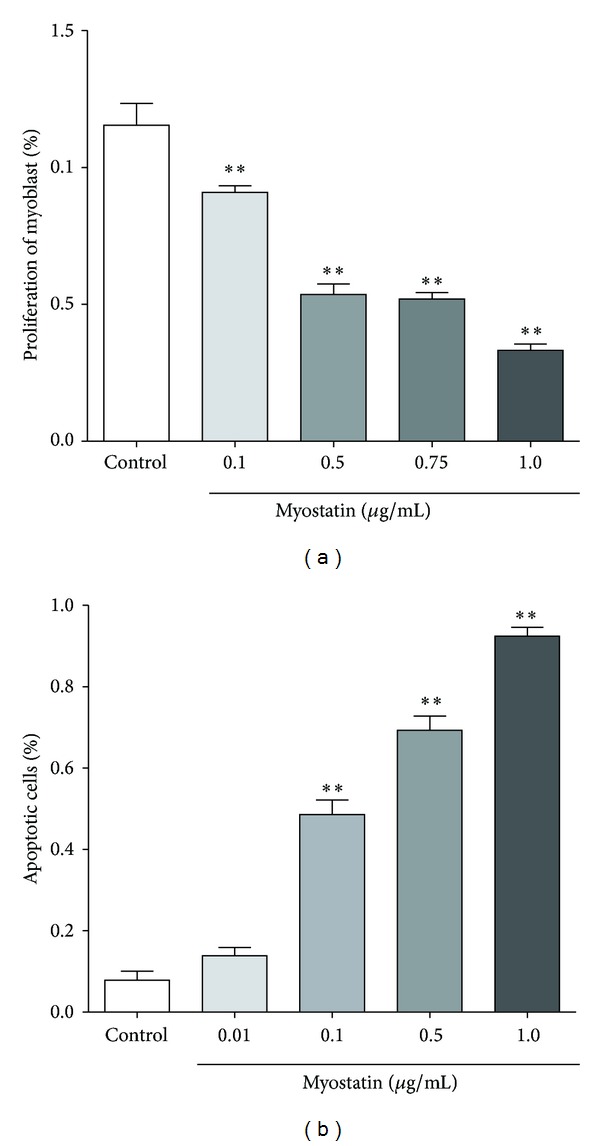
(a) Proliferation of murine myoblasts is shown as percentage after exposure to different myostatin concentrations for 24 h. Increasing concentrations of myostatin were associated with decreased proliferation rate of murine myoblasts. Myoblasts cultured with 1.0 *μ*g/mL myostatin for 24 h showed lowest proliferation (33.2%) in comparison to 0.75 *μ*g/mL (52.0%), 0.5 *μ*g/mL (53.6%), and 0.1 *μ*g/mL (90.87%) (*P < 0.001, in comparison to control group). (b) Apoptotic effect of myostatin on murine myoblasts is shown as percentage of apoptotic cells. Murine myoblasts exposed to 1.0 *μ*g/mL myostatin for 24 h showed highest apoptotic rate of 92.4% in comparison to 0.5 *μ*g/mL (69.3%), 0.1 *μ*g/mL (48.6%), and 0.01 *μ*g/mL (13.9%) (**P < 0.001, in comparison to control group).

To investigate the apoptotic effect of myostatin on myoblasts, increasing concentrations (0.01–1.0 *μ*g/mL) of murine myostatin were added to murine myoblasts and incubated for 24 h. Addition of 0.01 *μ*g/mL myostatin did not show a significant increase in apoptosis of murine myoblast cells. Murine myoblasts exposed to 1.0 *μ*g/mL myostatin for 24 h showed highest apoptotic rate of 92.4% in comparison to 0.5 *μ*g/mL (69.3%) and 0.1 *μ*g/mL (48.6%) (*P* < 0.001, in comparison to control group, Figure 4(a)). Proliferation of myoblasts was analyzed after 24 h myostatin exposure by performing a colorimetric BrdU-based ELISA. Addition of various myostatin concentrations resulted in a significant decrease (*P* < 0.001) of myoblasts proliferation whereas myoblasts cultured with 1.0 *μ*g/mL myostatin showed lowest proliferation (33.2%) in comparison to 0.75 *μ*g/mL (52.0%), 0.5 *μ*g/mL (53.6%), and 0.1 *μ*g/mL (90.87%) (<0.001, in comparison to control group, Figure 4(b)).

The concentration of 0.75 *μ*g/mL myostatin was selected for subsequent experiments due to significant effects on myoblasts apoptosis and proliferation.

### 3.4. IGF-1 Is Accountable for Protective Effect of ASCs Conditioned Medium

**Figure 5:**
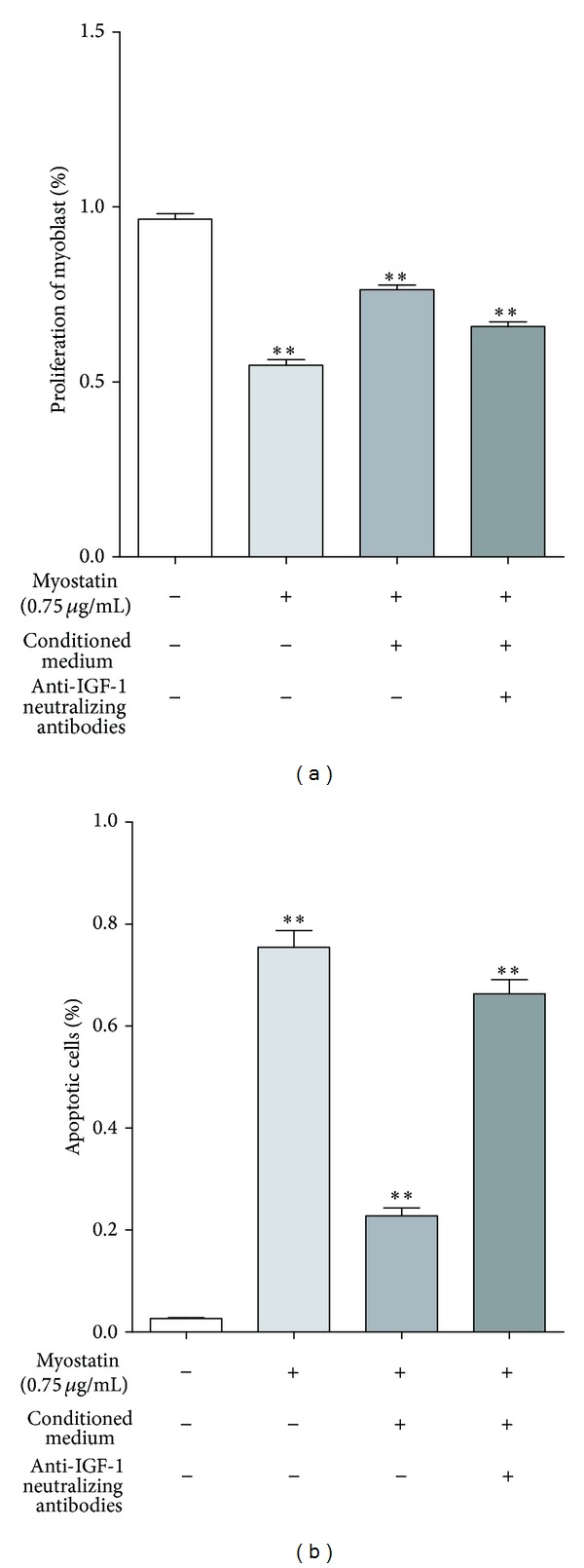
(a) Effect of conditioned medium on myoblasts' proliferation is shown as percentage after 48 h myostatin exposure. Proliferation of murine myoblast cultured in 0.75 *μ*g/mL myostatin significantly decreased to 54.8% (±3.2%, **P < 0.001), whereas conditioned medium of ASCs significantly increased myoblasts' proliferation under 0.75 *μ*g/mL myostatin incubation to 76.4% (±2.7%, **P < 0.001). This protective effect was abolished after neutralization of IGF-1 ligand-receptor interaction and proliferation significantly decreased to 65.8% (±2.9%, **P < 0.001). (b) Myoblasts exposed to 0.75 *μ*g/mL myostatin showed a significant increase in apoptosis rate to 75.5% (±8.7%) but were reduced to 22.8% (±4.2%) due to paracrine factors of ASCs conditioned medium (**P < 0.001). The protective effect of ASCs conditioned medium was abolished after blocking IGF-1 and its receptor whereas apoptotic rate increased to 66.3% (±7.3%) (**P < 0.001). Thus, IGF-1 secreted by ASCs accounts for 82.5% of the antiapoptotic effect of the CM (*N* = 3, *n* = 3).

Paracrine factors of ASCs were investigated for their effect on myoblasts' proliferation exposed to myostatin. The proliferation decreased by myostatin to 54.8% (±3.2%) was significantly abrogated by stem cell conditioned medium and increased to 76.4% (±2.7%) (Figure 5(a)). Neutralization antibodies against IGF-1 and its corresponding receptor were simultaneously added to conditioned medium in order to elucidate the protective role of IGF-1 secreted by ASCs. Proliferation of myoblasts decreased from 76.4% (±2.7%) to 65.8% (±2.9%) after blocking IGF-1 ligand-receptor interaction. Thus, IGF-1 secreted by ASCs accounts for ~51% of the protective effect on myoblasts proliferation when exposed to myostatin.

Furthermore, CM of ASCs reduced apoptosis rate of myostatin treated myoblasts from 75.5% (±8.7%) to 22.8% (±4.2%). But, after blocking the effect of IGF-1 in conditioned medium of ASCs the apoptosis rate significantly increased to 66.3% (±7.3%) (*P* < 0.001, Figure 5(b)). Thus, 82.5% of the antiapoptotic effect of the CM is due to IGF-1 secreted by ASCs.

### 3.5. IGF-1 Restores MyoD Expression in Myoblasts

**Figure 6:**
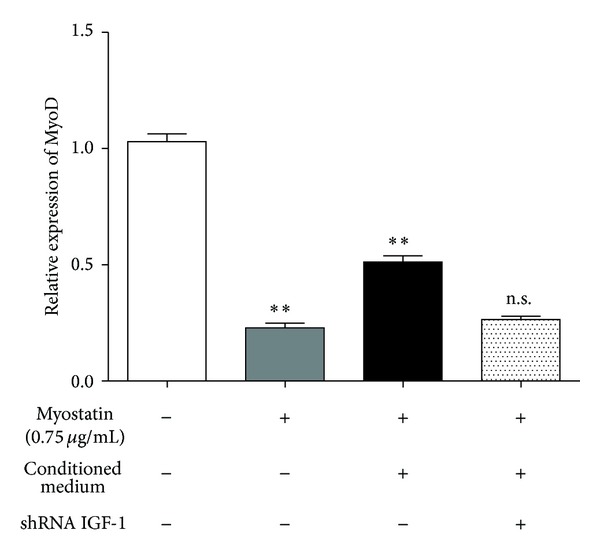
Relative quantification of MyoD mRNA is shown as mean ± SD percentage of MyoD mRNA expression in murine myoblasts after 72 h myostatin treatment. Myostatin exposure significantly reduced MyoD mRNA to 22.8% (±4.6%, **P < 0.001) in comparison to untreated myoblasts. Due to the effect of conditioned medium from wild type ASCs MyoD, mRNA significantly increased to 51.2% (±5.9%, **P < 0.001) when compared to myostatin treatment. But this effect was accountable for IGF-1 by 87.3% since IGF-1 depleted CM decreased MyoD mRNA level to 26.4% (±3.4%, n.s. compared to myostatin treatment) (*N* = 3, *n* = 3).

MyoD expression at mRNA and protein level was investigated to evaluate the influence of IGF-1 on murine myoblast transdifferentiation. First, the expression of IGF-1 was suppressed by a lentiviral shRNA vector construct in ASCs and efficiency was assessed by quantitative real-time PCR (97%) and ELISA (85%). ASCs showed a high expression of IGF-1 protein (328.33 ± 22.7 pg/*μ*g DNA) which was not affected by nontarget shRNA transfection. MyoD mRNA expression in myoblasts treated with 0.75 *μ*g/mL myostatin for 72 h decreased to 22.8% (±4.6%) in comparison to untreated myoblasts. Conditioned medium of wild type ASCs could partially abolish the negative effect of myostatin since MyoD mRNA significantly increased to 51.2% (±5.9%). This positive effect was accountable for IGF-1 by 87.3% since IGF-1 depleted CM decreased MyoD mRNA level to 26.4% (±3.4%) (Figure 6).

**Figure 7:**
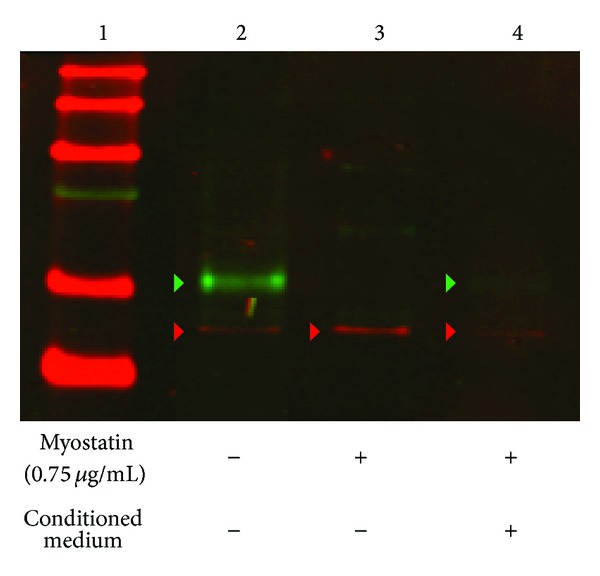
Representative Western blot analysis of MyoD protein expression. Nontreated cells clearly showed an expression of MyoD protein (lane 2) whereas no MyoD protein was detected after incubation with myostatin (lane 3). After incubation with CM of ASCs a weak but significant expression of MyoD was detectable by Western blot analyses (lane 4). A total protein amount of 90 *μ*g was loaded on each lane. Green arrow indicates the band of MyoD and red arrow indicates *β*-Actin as loading control (*N* = 3).

The expression of MyoD protein was examined in myoblast cells under 0.75 *μ*g/mL myostatin exposure with and without conditioned medium. Control samples clearly showed MyoD protein expression whereas protein was no longer detectable after myostatin treatment. But, addition of conditioned medium to myoblasts under myostatin treatment demonstrated a weak but distinct detection of MyoD protein expression (Figure 7).

## 4. Discussion

The aim of the study was to examine the regenerative potential of ASCs mediated by the secretion of paracrine factors particularly IGF-1 for dystrophic muscle diseases considering myostatin as a negative regulator of skeletal muscle mass.

Stem cell based therapy provides a promising treatment option for DMD since fusion between healthy stem cells and dystrophic myoblasts restored dystrophin expression* in vitro* and* in vivo* [12, 13]. We confirmed previous results that dystrophic myoblasts fuse with ASCs but clearly provide the evidence that nuclei from both cell types are apparent in emerged myotubes. The nucleus and the cell membrane of one cell line were labeled with two different fluorescent proteins in the present study and were monitored in real time. This represents a method superior to a single fluorescent cell labeling approach for fusion since currently used dyes (e.g., DAPI, Dio, Dil, DiD, quantum dots) provide only transient staining restricted to specific cell organelles (i.e., nucleus, cytoplasm, or mitochondria).

Fusion is a feature of myogenic precursor cells occurring after activation of muscle satellite cells in order to restore affected muscle tissue. But tremendous destruction of muscle cells reduces the capacity of satellite cells regarding proliferation and self-renewal [14]. Thus, application of stem cells provides muscle tissue with a cell population presenting intact self-renewal and proliferation potential. More recently, it has been shown that mouse adipose tissue stem cells restore dystrophin expression in mdx mice [12] but only at the site of injection [15]. Previous studies and our results provide evidence that ASCs possess the capacity to contribute to muscle regeneration by gene expression modification after fusion. However, the paracrine effects of ASCs have not been investigated on dystrophic myoblast as previously for myocardial infarction [11] or hind limb ischemia [16]. The major finding of this study is that IGF-1 secreted by ASCs increase MyoD expression in myostatin treated myoblasts. Furthermore, the paracrine factors of ASCs, particularly IGF-1, provide protective effects on murine myoblasts under myostatin treatment.

IGF-1 plays an essential role in muscle development, muscle cell proliferation, and muscle growth as already reported for insulin [17, 18] due to its structural resemblance. However, the anabolic function of IGF-1 is based on the interaction between binding proteins and IGF-1 receptor in an autocrinal and hormonal manner [19].

In contrast, TGF-*β* acts as an antagonist of IGF-1 by using the same signaling cascade (PI3K/Akt/GSK-3*β* pathway) [20]. This is of interest, since previous studies indicated an elevated TGF-*β* protein level in dystrophic muscles [7, 8]. Noteworthy, myostatin belongs to the TGF-*β* family and is known as an inhibitor of muscle growth and myoblasts' proliferation [21, 22]. In line, *in vitro* studies provided evidence that application of recombinant myostatin [23] or myostatin transfected myoblasts [24] was associated with decreased proliferation of muscle cells. Recently, it was shown that dystrophic muscle disease symptoms decreased in mdx mice when treated with myostatin antibodies [9].

These findings indicate that myostatin is involved in degeneration of skeletal muscles in DMD and that IGF-1 might be able to improve the inhibitory effect of myostatin on muscle growth. We for the first time could demonstrate that IGF-1 secreted by ASCs improves proliferation and viability of myostatin treated myoblasts. In line, hypertrophic effects on skeletal muscles were demonstrated *in vivo* for IGF-1 overexpressing mouse [25]. Moreover, muscle hypertrophy due to exercise is mediated by upregulation of IGF-1 mRNA [26]. But excessive physical muscle activity is critical for DMD patients and results in muscle fiber damage.

Gregorevic et al. [27] showed an improvement of muscle fatigue in EDL (extensor digitorum longus) and soleus muscles in IGF-1 treated mdx mice. IGF-1 is able to activate muscle growth and hypertrophy and seems to ameliorate the loss of muscle mass in DMD. However, growth factors have a limited half-life and are small in size, which restricts their retention within a tissue for prolonged periods. Moreover, systemic administration of growth factors has shown a modest success or unpleasant side effects in a number of *in vivo* studies even when using gene transfer [23–26]. For this reason, cell based therapy is advantageous since mesenchymal stem cells produce antiapoptotic factors at a bioactive level [7].

Activation of muscle differentiation requires myogenic regulatory factors (MRFs) consisting of four transcription factors including MyoD, myogenin, Myf5, and MRF4 [28, 29]. Of importance, quiescent satellite cells express no detectable levels of MRFs and first express MyoD or Myf-5 after activation. Regeneration in MyoD−/− muscle is characterized by almost complete abolished proliferation of myogenic cells [30]. In line, Langley et al. [31] provided evidence that MyoD expression is significantly decreased in myoblasts by myostatin treatment. Moreover, MyoD can be downregulated by myostatin treatment even after initial induction due to myogenic differentiation. In addition, the study revealed that myostatin treatment increased Smad3 level that coimmunoprecipitated with MyoD suggesting Smad3 binding to MyoD thereby inhibiting muscle differentiation. IGF-1 has been reported to block Smad3 activation via a phosphatidylinositol 3-kinase (PI3K)/Akt dependent pathway and thereby inhibiting TGF-*β* signaling [32]. Moreover, Trendelenburg et al. reported that myostatin inhibits the activation of Akt signaling and IGF-1 treatment can counteract myostatin's antidifferentiation effects [33]. These previous results support our findings that IGF-1 secreted by ASCs can restore the negative effect of myostatin on MyoD expression.

## 5. Conclusion

The present study indicates that IGF-1 secreted by ASCs improves the catabolic effect of myostatin on myoblasts. In addition, our results support that ASCs might represent a valuable cell source for local application in DMD treatment due to their high secretion of bioactive IGF-1. These results show the important paracrine action of adipose tissue derived stem cells in therapy for muscular dystrophies and muscle wasting diseases.

## Figures and Tables

**Table 1:** Primer sets for SYBR Green assay.

Primer	Tm		Sequence
Actin	55°C	Sense	CAGGTCCAGACGCAGGATGGC
		Antisense	CTACAATGAGCTGCGTGTGGC

MyoD	57°C	Sense	GGTCTGGGTTCCCTGTTCTGTGT
		Antisense	CCCCGGCGGCAGAATGGCTACG

GAPDH	67°C	Sense	AGCCACATCGCTCAGACACC
		Antisense	GTACTCAGCCGCCAGCATCG

IGF-1	60°C	Sense	GCTGGTGGAAGCTCTTCAGTTC
		Antisense	AGCTGACTTGGCAGGCTTGAG
